# Protocol for evaluating humoral immune responses in mice following SARS-CoV-2 vaccination

**DOI:** 10.1016/j.xpro.2024.103548

**Published:** 2025-01-15

**Authors:** Yao Zhang, Shixiong Li, Jingyou Yu

**Affiliations:** 1 Guangzhou National Laboratory, Bio-Island, Guangzhou, Guangdong 510005, China; 2 Zhongshan School of Medicine, Sun Yat-sen University, Guangzhou, Guangdong 510080, China; 3 College of Life Sciences, Nankai University, Tianjin 300071, China; 4 State Key Laboratory of Respiratory Disease, The First Affiliated Hospital of Guangzhou Medical University, Guangzhou, Guangdong 510182, China

**Keywords:** cell biology, cell-based assays, immunology, microbiology, molecular biology

## Abstract

Binding and neutralizing antibodies are critical indicators of protection against viral pathogens and are essential for assessing the immunogenicity and efficacy of a vaccine. Here, we present a protocol comprising two assays for measuring the spike-specific binding and neutralizing antibodies in mouse plasma following severe acute respiratory syndrome coronavirus 2 (SARS-CoV-2) vaccination. We describe steps for determining binding antibody titers using enzyme-linked immunosorbent assay (ELISA) and assessing neutralizing antibody titers through a pseudovirus neutralization assay.

For complete details on the use and execution of this protocol, please refer to Jingyou Yu et al.^1^

## Highlights

• Produce and purify an adenoviral-vectored vaccine for mouse immunization
• Measure binding antibody titers using ELISA from immunized mouse plasma
• Evaluate neutralizing antibody titers with a pseudovirus neutralization assay

## Abstract (editor highlights)

Publisher’s note: Undertaking any experimental protocol requires adherence to local institutional guidelines for laboratory safety and ethics.

## Graphical abstract

**Figure:**
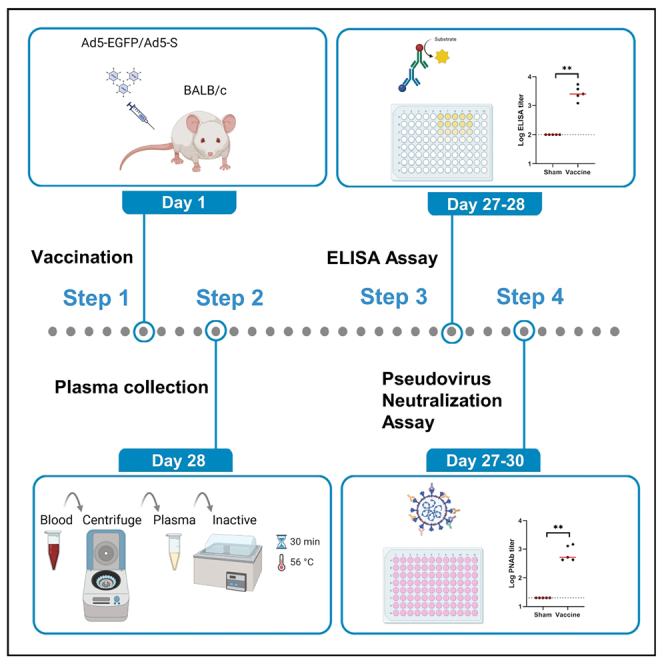

## Before you begin

### Institutional permission

This protocol involves the use of female BALB/c mice aged 6–8 weeks. All experiments must be conducted in accordance with the protocol approved by the Institutional Animal Care and Use Committee (IACUC) and in compliance with state and institutional regulations. These experimental procedures are certified by the AAALAC and conducted in a Specific Pathogen Free (SPF) laboratory animal facility.

### Preparation 1: Adenoviral-vectored vaccine production

**Timing: 1–2weeks(for step 1)**

**Timing: 18–36 days (for step 2)**

**Timing: 4–6 days (for step 3a)**

**Timing: 4–6 days (for step 3b)**

**Timing: 2 days (for steps 4–8)**

This step focuses on the production and purification of the adenoviral vectored vaccine in preparation for mouse immunization.

**Figure 1:**
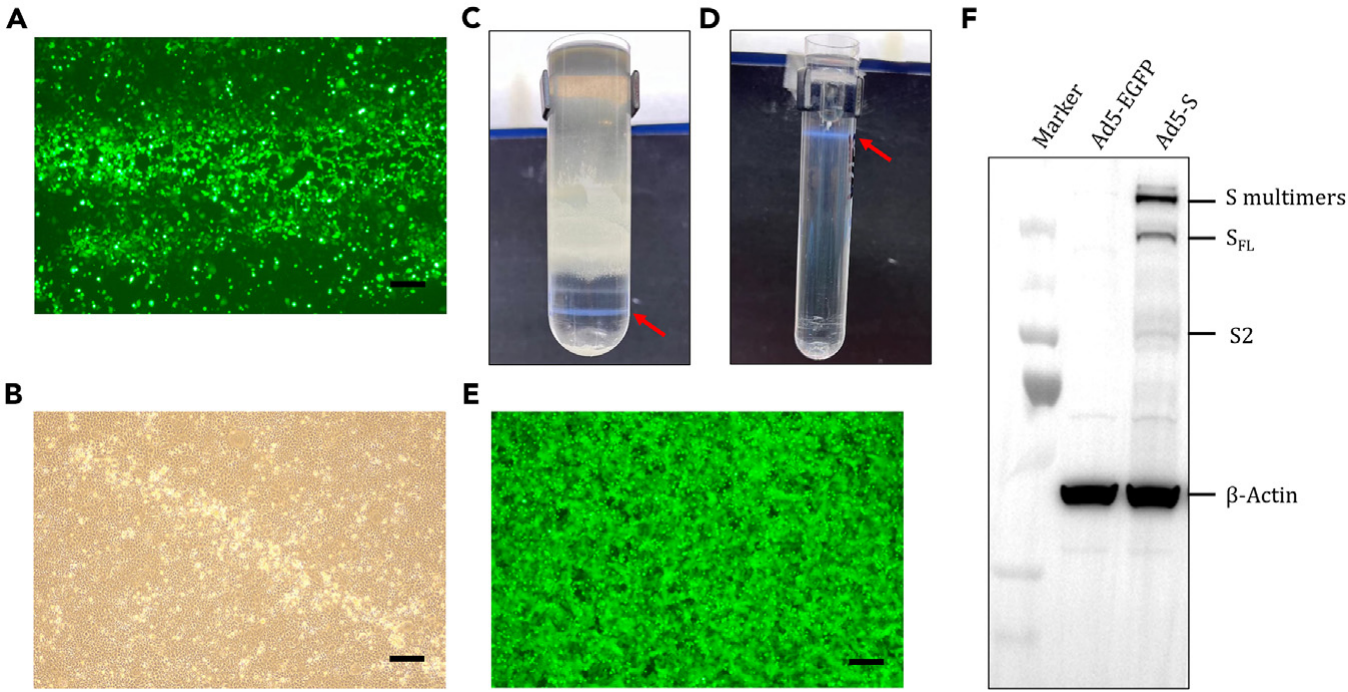
Production, purification, and expression of adenoviral vectored vaccines After transferring the HEK 293A cells packaging adenovirus to a T75 flask, CPE are observed between days 5 and 14. (A) Ad5-EGFP, (B) Ad5-S. (C) CsCl Gradient Purification (Block Gradient). (D) CsCl Gradient Purification (Continuous Gradient). The red arrow indicates the adenovirus band. (E) Fluorescence microscopy of A549 cells infected by Ad5-EGFP after 48 h. (F) Western blot (WB) analysis detecting spike protein expression in A549 cells 48 h post-infection with Ad5-EGFP/S viruses. Scale bars represent 200 μm.

1. Clone the target gene encoding the spike protein of SARS-CoV-2 prototype strain WA01/2020 or EGFP into the pBR322-based adaptor plasmid pAdApt, using the Hind III and EcoR I restriction sites, driven by the cytomegalovirus (CMV) immediate-early promoter and terminated by an SV40 polyadenylation signal.
2. Adenovirus production.
  a. Transfection Preparation.
    Day 1.
    i. Seed 2.5×10^6^ HEK293A cells into T25 flasks.
      Day 2.
    ii. Digest 2 μg of pAdApt and 6 μg of pWE plasmids using PacI restriction enzyme. Incubate at 37°C for 2–3 h.
      ***Note:*** After incubation, run 2 μL of the sample on an electrophoresis gel to verify complete digestion and confirm the correct band sizes. Linearized plasmids can be stored at −20°C for a few days. Long-term storage is not recommended due to the low plasmids concentration in this system; any degradation could significantly affect subsequent steps.
    iii. Dilute the plasmids and 24 μg PEI each in 500 μL of Opti-MEM. Mix the diluted PEI with the diluted plasmids solution and incubate at 25°C for 15 min. Transfect the linearized plasmids into HEK293A cells in the T25 flask.
      ***Note:*** Ensure the cells are at 70%–80% confluence before transfection.
    iv. Incubate the flasks at 37°C with 5% CO_2_ for 48 h.
      Day 4.
  b. Use 0.05% trypsin-EDTA to detach the cells from the T25 flask. Transfer cells from the T25 flask to the T75 flask.
    Day 9–36.
  c. Observe for CPE (Cytopathic Effect) (Figures 1A and 1B) between 5 to 14 days after transfer.
    ***Note:*** After adenovirus replication, cytopathic effects (CPE) can be observed. In the early stages, a few cells exhibit rounding and detachment under the microscope, with a comet-tail appearance visible. As CPE progresses to full CPE, all cells become rounded and detached, though lysis has not yet occurred.
  d. Once the CPE is evident, with most cells rounded and detached but not yet lysed, use a cell scraper to collect all the cells. The resulting mixture of cells and culture medium is referred to as the “crude culture.” Transfer the crude culture into a 15 mL tube for further processing.
  e. Perform three freeze (−20°C) and thaw (37°C) to release the virus.
    **CRITICAL:** If full CPE is observed, harvest the crude culture into a 15 mL tube and freeze at −20°C, then thaw at 37°C, repeating the freeze-thaw cycle three times. If partial CPE is observed, follow the same harvesting and freeze-thaw process.
    Subsequently, centrifuge the crude lysate at 500 *g* for 5 min at 25°C and remove 2 mL of the supernatant to re-infect a T75 flask of HEK293A cells. If no CPE is observed by the time the cells die from overcrowding (approximately Day 14), harvest the crude culture and centrifuge as previously described, preparing for re-infection. If no full CPE is observed after two rounds of reinfection, proceed with re-transfection.
    **Pause point:** Store crude culture at −80°C for long-term storage.
3. Adenovirus expansion.
  a. Expand Working Virus Seed Stock (WVSS).
    Day 1.
    i. Seed 2×10^7^ HEK 293A cells into the T175 flask.
      Day 2.
    ii. Add 2 mL of viral stock into the T175 flask to infect the HEK 293A cells.
      ***Note:*** Ensure the cells reach 70%–80% confluence before viral infection.
      Day 4∼6.
    iii. Monitor for CPE. Harvest the crude cell culture on the day full CPE is observed. Collect the culture in a 50 mL tube, freeze at −20°C, and then thaw at 37°C, repeating this freeze-thaw process three times. Centrifuge the crude lysate at 500 *g* for 5 min, and collect the supernatant, approximately 25 mL, to serve as the WVSS.
      **Pause point:** Store WVSS at −80°C for long-time storage.
  b. Adenovirus expansion.
    Day 1.
    i. Seed HEK 293A cells into five 5-layer T175 flasks.
      Day 2.
    ii. Inoculate each 5-layer T175 flask with 4 mL of WVSS.
      ***Note:*** Ensure the cells reach 70%–80% confluence before viral infection.
      Day 4∼6.
    iii. Monitor for CPE. Harvest the cells and culture medium once complete CPE is observed. Collect in a 50 mL centrifuge tube, centrifuge at 500 *g* for 5 min, discard the supernatant, and transfer the cells into six 50 mL centrifuge tubes.
    iv. Add 19 mL of 1× PBS to each tube and resuspend cells by pipetting.
    v. Freeze the tubes at −20°C.
      **Pause point:** Store tubes at −80°C for long-term storage.
4. Purification of adenoviral vectored vaccine.
  Day 1.
  a. Lyse the cells.
    i. Thaw the frozen production at 37°C until completely thawed.
    ii. Add 2.25 mL of 5% sodium deoxycholate (DOC) to each tube to achieve a final concentration of 0.5% DOC. Homogenize the suspension by inverting 20 times.
      ***Note:*** Avoid shaking; the mixture should appear thick and viscous.
    iii. Incubate 37°C for 5 min, then homogenize by inverting 20 times.
  b. Clarification.
    i. Add 750 μL of 1 M MgCl_2_ per tube and homogenize by inverting 20 times.
      ***Note:*** Avoid shaking; a milky white precipitate should form.
    ii. Add 80 μL 10 mg/mL DNase and homogenize by inverting 20 times.
      ***Note:*** Avoid shaking.
    iii. Incubate at 37°C for 15 min.
      ***Note:*** A significant decrease in viscosity should be observed.
    iv. Homogenize by inverting 20 times, then centrifuge at 12,000 *g* for 30 min to remove cell debris.
      ***Note:*** Pour off the supernatant during this spin.
    v. Transfer supernatant to new tubes.
  c. CsCl Gradient Purification (Block Gradient).
    i. Prepare the CsCl solution: Add 10 mL of 1.24 g/mL CsCl to ultracentrifuge tubes. Using a Pasteur pipette, carefully layer 5 mL of 1.4 g/mL CsCl at the bottom, followed by 23–25 mL of the viral supernatant.
      **CRITICAL:** Ensure the pipette touches the wall of the centrifuge tube to avoid disturbing the gradient.
    ii. Balance the tubes to an accuracy of 0.01 g.
    iii. Ultracentrifuge using an AH-629 swinging bucket rotor at 21,000 rpm (58,400 g) for 2 h at 10°C.
    iv. Prepare a stand with clamp and black background over bucket of 10% SDS.
    v. Use a 0.9–40 mm needle and 10 mL syringe to puncture the tube wall at an upward angle and carefully extract the viral band into a new tube.
      ***Note:*** White or blue virus band should be visible between 1.24 and 1.4 g/mL layers (Figure 1C). No bands should be found beneath the virus band, while the band above contains empty capsids, and the one above that contains hexon protein.
  d. CsCl Gradient Purification (Continuous Gradient).
    i. Transfer the collected viral liquid into 13.2 mL ultracentrifuge tubes, adding up to 7 mL in each. Add 1.33 g/mL CsCl, balance the tubes, and ultracentrifuge using a TH-641 swinging bucket rotor at 55,000 rpm (260,000 g) at 4°C for 10–18 h.
      Day 2.
    ii. Prepare a stand with clamp and black background over bucket of 10% SDS.
    iii. Use a 0.9–40 mm needle and 10 mL syringe to puncture the tube wall at an upward angle and carefully extract the viral band to a new tube.
    iv. Extract lowest virus band (Figure 1D) with the needle and syringe, aiming to minimize the liquid volume collected.
      ***Note:*** The virus band is located in the middle of the tube; the upper bands contain empty capsids and hexon protein.
5. Dialysis.
  a. Prepare dialysis buffer 1, 2 and 3.
  b. Use a clean, autoclaved 3 L beaker with stir bar in hood, add 1.5 L dialysis buffer 1.
  c. Place the CsCl-virus mixture into a dialysis membrane and secure it with dialysis clips.
    ***Note:*** The dialysis membrane has a 10 kDa cutoff and is suitable for a sample volume of 3–15 mL.
  d. Place the dialysis membrane into a beaker containing dialysis buffer 1 and stir at 200 rpm at 25°C for 1 h of dialysis.
  e. Replace buffer 1 with buffer 2 and continue dialysis for an additional hour.
  f. Replace buffer 2 with buffer 3 and continue dialysis for the final hour.
  g. Transfer the virus into a 50 mL tube.
6. Aliquot the appropriate volume of purified virus into 1.5 mL Eppendorf tubes and store at −80°C for long-term storage.
7. Use the primer pair pAdapt-PF and pAdapt-PR to perform qPCR for determining the viral particle (vp) titers.
8. Infect the A549 cell line with purified adenovirus at a dose of 5 MOI. After 48 h, observe EGFP protein expression under a fluorescence microscope (Figure 1E) and verify S protein expression by Western blotting (Figure 1F).

***Note:*** All items that come into direct contact with the adenovirus during these steps must be wiped or soaked with 1% SDS to inactivate the virus.

### Preparation 2: Mice immunization

**Timing: 4 weeks**

This step mainly involves immunizing BALB/c mice with adenovirus-vectored vaccines via intramuscular injection to evaluate the humoral immune response.

9. Maintain specific pathogen-free 6- to 8-week-old BALB/c mice in the Animal Care Facilities, randomly allocating 5 mice per cage.
10. Immunize the mice with 1 × 10^8^ vp adenoviral-vectored vaccines in a total volume of 100 μL, administered through bilateral intramuscular injection into the quadriceps muscles (Table 1).

***Note:*** Avoid repeated freezing and thawing of vaccines.

11. Four weeks after the vaccination, collect 100–200 μL peripheral blood via the submandibular route and isolate plasma for immunological assays by centrifuging at 1,000 *g* at 25°C for 10 min.

**Pause point:** Plasma can be frozen and stored at −80°C for long-term use.

**Table 1:** Ad5-vectored vaccines preparation for mice

Group vaccine		Concentration	Total volume	Mice
1	Ad5-EGFP	1 × 10^9^ vp/mL	500 μL	5
2	Ad5-S	1 × 10^9^ vp/mL	500 μL	5

## Key resources table

**Table:** 

REAGENT or RESOURCE	SOURCE	IDENTIFIER
**Antibodies**		

Goat anti-mouse IgG-HRP (1:4,000)	Sino Biological	Cat# SSA007

**Biological samples**		

Mice plasma	This paper	N/A

**Chemicals, peptides, and recombinant proteins**		

PacⅠ	NEB	Cat# R0547
Sodium deoxycholate (DOC)	Sigma	Cat# D6750
1 M MgCl_2_	LEAGENE	Cat# R00482
DNaseⅠ	Roche	Cat# 104159
CsCl	Macklin	Cat# C804679
MgCl_2_·6H_2_O	Sigma	Cat# M2393
CaCl_2_·2H_2_O	Sigma	Cat# C7902
Sucrose	Macklin	Cat# S818046
1 M Tris HCl pH 7.5	Yuanye	Cat# R21385
Glycerol	Macklin	Cat# G810575
Imidazole	Solarbio	Cat# 288-32-4
Protease inhibitor cocktail	MCE	Cat# HY-K0010
Amicon Ultra column	MCE	Cat# UFC9030
ELISAcoating buffer	Solarbio	Cat# C1050
Difco Skim milk	BD Life Sciences	Cat# 90002-594
Tween 20	Diamond	Cat# A100777- 0500
TMB	Solarbio	Cat# PR1200
ELISA Stop buffer	Solarbio	Cat# C1058
PEI	Polysciences	Cat# 23966
CD 293 TGE medium	ACROBiosystems	Cat# CM-1156-11
High-affinity Ni-NTA resin	GenScript	Cat# L00250
1 × PBS	Biosharp	Cat# BL302A
0.05% trypsin-EDTA	Gibco	Cat# 25300062
DMEM	Gibco	Cat# C11995500BT
FBS	ExCell Bio	Cat# FSP500
Opti-MEM I reduced serum medium	Gibco	Cat# 31985070
Firefly Luciferase Reporter Gene Assay Kit	Beyotime	Cat# RG006

**Experimental models: Cell lines**		

HEK 293A	This paper	N/A
A549	This paper	N/A
HEK 293F	This paper	N/A
HEK 293T	ATCC	Cat# CRL-11268
HEK 293T-ACE2	This paper	N/A

**Experimental models: Organisms/strains**		

BALB/c mice, 6–8 weeks old, female	Beijing Vital River Laboratory Animal Technology Co., Ltd.	N/A

**Oligonucleotides**		

pAdApt-PF	GCCGGGAACGGTGC ATTGGA	N/A
pAdApt-PR	GAGGATCCGTTAAC GCTAGC	N/A

**Recombinant DNA**		

pcDNA3.1-spike RBD	This paper	N/A
PsPAX2	Addgene	Cat# 12260
pLenti-CMV Puro-Luc	Addgene	Cat# 17477
pcDNA3.1-S.dCT13	This paper	N/A
pWE.Ad.AflII-rITR.dE3	This paper	N/A
pAdApt-EGFP	This paper	N/A
pAdApt-S WA01/2020	This paper	N/A

**Software and algorithms**		

GraphPad Prism 9.0.0	GraphPad Software, Inc.	https://www.graphpad.com/

**Other**		

T25 flask	NEST	Cat# 707003
T75 flask	NEST	Cat# 708003
T175 flask	NEST	Cat# 709003
5-layer flask	NEST	Cat# 731002
38.5 mL ultracentrifuge tube	Beckman	Cat# 344058
13.2 mL ultracentrifuge tube	Beckman	Cat# 344059
10 kDa cut-off dialysis membrane	Yuanye	Cat# SP131276
Sealing film	Solarbio	Cat# YA0247
96-well tissue culture plate	NEST	Cat# 701001
96-well EIA/RIA plate	Corning	Cat# 3590
96-well round bottom plate	Corning	Cat# 3799
White plate	Corning	Cat# 3922
High-affinity Ni-NTA resin	GenScript	Cat# L00250

## Materials and equipment

**Table:** Digestion of pAdApt

Regent	Amount	Volume
pAdApt	2 μg	X μL
10 × rCutSmart buffer	N/A	3 μL
PacI	20 U	2 μL
ddH_2_O	N/A	25-X
**Total**	**N/A**	**30 μL**

***Note:*** After digestion, heat inactivate and sterilize at 65°C for 30 min.

**Table:** Digestion of pAdApt

Regent	Amount	Volume
pWE	6 μg	X μL
10 × rCutSmart buffer	N/A	3 μL
PacI	60 U	6 μL
ddH_2_O	N/A	21-X
**Total**	**N/A**	**30 μL**

***Note:*** After digestion, heat inactivate and sterilize at 65°C for 30 min.

**Table:** 5% DOC

Regent	Amount	Volume	Concentration
Sodium deoxycholate (DOC)	5 g	N/A	5%
ddH_2_O	N/A	100 mL	N/A
**Total**	**N/A**	**100 mL**	**N/A**

***Note:*** Sterilized by filtration. Store at RT for 1 year.

**Table:** 10 mg/mL DNase I

Regent	Amount	Volume	Concentration
DNase I	100 mg	N/A	10 mg/mL
ddH_2_O	N/A	10 mL	N/A
**Total**	**N/A**	**10 mL**	**N/A**

***Note:*** Store at −20°C for 1 year.

**Table:** 1.2 g/mL CsCl, 5% v/v glycerol in 25 mM Tris pH 7.5

Regent	Amount	Volume
CsCl	278 g	N/A
1 M Tris HCl pH 7.5	N/A	30 mL
Glycerol	N/A	50 mL
ddH_2_O	N/A	920 mL
**Total**	**N/A**	**1000 mL**

***Note:*** Dissolve 278 g CsCl in 700 mL ddH_2_O, add 30 mL 1 M Tris HCl pH 7.5 and 50 mL glycerol, add ddH_2_O to 1000 mL, weigh 1 mL solution, adjust weight to 1.2 g by adding CsCl or ddH_2_O. Store at RT for 1 year.

**Table:** 1. 4 g/mL CsCl, 5% v/v glycerol in 25 mM Tris pH 7.5

Regent	Amount	Volume
CsCl	478 g	N/A
1 M Tris HCl pH 7.5	N/A	30 mL
Glycerol	N/A	50 mL
ddH_2_O	N/A	920 mL
**Total**	**N/A**	**1000 mL**

***Note:*** Dissolve 478 g CsCl in 700 mL ddH_2_O, add 30 mL 1 M Tris HCl pH 7.5 and 50 mL glycerol, add ddH_2_O to 1000 mL, weigh 1 mL solution, adjust weight to 1.4 g by adding CsCl or ddH_2_O. Store at RT for 1 year.

**Table:** 1.24 g/mL CsCl, 5% v/v glycerol in 25 mM Tris pH 7.5

Regent	Amount	Volume
CsCl	318 g	N/A
1 M Tris HCl pH 7.5	N/A	30 mL
Glycerol	N/A	50 mL
ddH_2_O	N/A	920 mL
**Total**	**N/A**	**1000 mL**

***Note:*** Dissolve 318 g CsCl in 700 mL ddH_2_O, add 30 mL 1 M Tris HCl pH 7.5 and 50 mL glycerol, add ddH_2_O to 1000 mL, weigh 1 mL solution, adjust weight to 1.24 g by adding CsCl or ddH_2_O. Store at RT for 1 year.

***Alternative:*** 800 mL 1.2 g/mL CsCl plus 200 mL 1.4 g/mL CsCl.

**Table:** 1.33 g/mL CsCl, 5% v/v glycerol in 25 mM Tris pH 7.5

Regent	Amount	Volume
CsCl	408 g	N/A
1 M Tris HCl pH 7.5	N/A	30 mL
Glycerol	N/A	50 mL
ddH_2_O	N/A	920 mL
**Total**	**N/A**	**1000 mL**

***Note:*** Dissolve 408 g CsCl in 700 mL ddH_2_O, add 30 mL 1 M Tris HCl pH 7.5 and 50 mL glycerol, add ddH_2_O to 1000 mL, weigh 1 mL solution, adjust weight to 1.33 g by adding CsCl or ddH_2_O. Store at RT for 1 year.

***Alternative:*** 350 mL 1.2 g/mL CsCl plus 650 mL 1.4 g/mL CsCl.

**Table:** 50% sucrose

Regent	Amount	Volume	Concentration
Sucrose	250 g	N/A	50%
ddH_2_O	N/A	500 mL	N/A
**Total**	**N/A**	**500 mL**	**N/A**

***Note:*** Make up to 500 mL with ddH_2_O and autoclave at 121°C for 15 min. Store at RT for 1 year.

**Table:** 200 × Ca-MgCl_2_

Regent	Amount	Volume
MgCl_2_·6H_2_O	13.25 g	N/A
CaCl_2_·2H_2_O	10 g	N/A
ddH_2_O	N/A	500 mL
**Total**	**N/A**	**500 mL**

***Note:*** Autoclave at 121°C for 15 min. Store at RT for 1 year.

**Table:** Dialysis buffer 1

Regent	Volume
50% sucrose	30 mL
200 × CaMgCl_2_	7.5 mL
1 × PBS	1500 mL
**Total**	**1537.5 mL**

***Note:*** Prepare fresh.

**Table:** Dialysis buffer 2

Regent	Volume
50% sucrose	60 mL
200 × CaMgCl_2_	7.5 mL
1 × PBS	1500 mL
**Total**	**1567.5 mL**

***Note:*** Prepare fresh.

**Table:** Dialysis buffer 3

Regent	Volume
50% sucrose	90 mL
200 × CaMgCl_2_	7.5 mL
1 × PBS	1500 mL
**Total**	**1597.5 mL**

***Note:*** Prepare fresh.

**Table:** Block buffer

Regent	Amount	Volume	Concentration
Skim milk	0.5 g	N/A	5%
1 × PBS	N/A	10 mL	N/A
**Total**	**N/A**	**10 mL**	**N/A**

***Note:*** Prepare fresh.

**Table:** Wash buffer

Regent	Volume	Concentration
Tween 20	0.5 mL	0.05%
1 × PBS	1000 mL	N/A
**Total**	**1000 mL**	**N/A**

***Note:*** Store at RT for 1 week.

## Step-by-step method details

### Spike protein production and purification

**Timing: 7–8 days**

This step mainly involves the production and purification of the Spike RBD protein, which is used to measure antibody titers in the plasma of immunized mice.

1. The RBD protein is produced in-house.
2. The codon-optimized DNA encoding SARS-CoV-2 RBD is artificially synthesized and subcloned into a pcDNA3.1 vector with a 6 × His tag at the C-terminus using the HindIII and EcoRI restriction sites.
3. Seed HEK-293F cells at a density of 0.5 × 10^6^ cells/mL in 400 mL of CD 293 TGE Medium in a 1 L shaker flask.
4. Incubate for 24 h in an orbital shaker at 37°C, 150 rpm, and 5% CO_2_ until the cells reach a density of 2.0 × 10^6^ cells/mL with 95%∼98% viability.
5. Dilute 400 μg of filter-sterilized RBD plasmid DNA into 20 mL of CD 293 TGE Medium and vortex for 5 s.
6. Add 1.2 mL of filter-sterilized 1 mg/mL PEI solution to the plasmid DNA solution, vortex vigorously for 5 s, mix well, and incubate at 25°C for 20 min.
7. Transfer DNA/PEI mixed solution into 400 mL of HEK-293F cells and incubate the cells in an orbital shaker at 37°C with 5% CO_2_ for 5 days.
8. Harvest the culture medium by centrifugation at 12,000 *g* for 15 min and filter the supernatant through a 0.22 μm filter.
9. Adjust the pH of the supernatant to 7.4 using 1 M Tris-HCl (pH = 8) and then use a peristaltic pump to pass the medium through a His GraviTrap column while on ice.
10. Wash the column with 50 column volumes of wash buffer (1 × PBS contains 20 mM imidazole).
11. Elute the HisTrap column with 2 column volumes elution buffer (1 × PBS contains 100/200/300 mM imidazole, respectively).
12. Collect the high purity protein and exchange the buffer to 1 × PBS using a 10 kDa Amicon Ultra column.
13. Collect and concentrate the protein, then store it at −80°C.

### ELISA process

**Timing: 2 days**

This step focuses on measuring the antibody titers in the plasma of mice immunized with the adenoviral vector vaccine using an enzyme-linked immunosorbent assay (ELISA).^1^^,^^2^^,^^3^^,^^4^^,^^5^^,^^6^^,^^7^^,^^8^

**Table 2:** Plasma dilution for the ELISA assay

Sample ID	1	2	3	4	5	6	7	8	9	10	NC -
**A**	1:100	1:100	1:100	1:100	1:100	1:100	1:100	1:100	1:100	1:100	N/A
**B**	1:300	1:300	1:300	1:300	1:300	1:300	1:300	1:300	1:300	1:300	N/A
**C**	1:900	1:900	1:900	1:900	1:900	1:900	1:900	1:900	1:900	1:900	N/A
**D**	1:2700	1:2700	1:2700	1:2700	1:2700	1:2700	1:2700	1:2700	1:2700	1:2700	N/A
**E**	1:8100	1:8100	1:8100	1:8100	1:8100	1:8100	1:8100	1:8100	1:8100	1:8100	N/A
**F**	1:24300	1:24300	1:24300	1:24300	1:24300	1:24300	1:24300	1:24300	1:24300	1:24300	N/A
**G**	1:72900	1:72900	1:72900	1:72900	1:72900	1:72900	1:72900	1:72900	1:72900	1:72900	N/A
**H**	1:218100	1:218100	1:218100	1:218100	1:218100	1:218100	1:218100	1:218100	1:218100	1:218100	N/A

NC for negative control.

Day 1.

14. Coat ELISA plates.
  a. Thaw the RBD protein, mix gently, and dilute in 1 × PBS to a final concentration of 1 μg/mL for coating 96-well ELISA plates.
  b. Using a multichannel pipette, add 100 μL of diluted protein to each well, ensuring to cover the bottom of the plate thoroughly. Cover the plates with plastic covers and store 12 h at 4°C.

***Note:*** Slightly increase the volume if necessary, when using the multichannel pipette.

Day 2.

15. Block ELISA plates.
  a. Prepare at least 10 mL block buffer per plate.
  b. Wash the coated ELISA plates three times with 300 μL of 0.05% PBST using a 96-well dispenser.
  c. Add 100 μL of blocking solution to each well and incubate at 25°C for 2 h shaking at 100 rpm.
16. Pre-dilute plasma.
  a. Set up 96-well round-bottom cell culture plates for 3-fold serial dilution of samples.
  b. Using a multichannel pipettor, add 120 μL of PBS containing 1% milk to all wells, and add an additional 78 μL to the wells in line A only.
  c. Add 2 μL plasma to line A and pipette up and down four to six times to mix.
    ***Note:*** The dilution ratio for plasma samples in line A is 1:100.
  d. Transfer 60 μL (3-fold dilution) from line A to line B and mix well. Repeat this process through line H, discarding 60 μL from line H (Table 2).
17. Incubate samples and secondary antibody (HRP).
  a. After blocking, discard the blocking solution and wash the plates twice with 200 μL 0.05% PBST.
  b. Add 100 μL sample to the corresponding wells in order, from the lowest to the highest concentration.
  c. Seal the plates and incubate for 2 h at 25°C.
  d. After incubation, wash the plates 5 times with 200 μL of 0.05% PBST.
  e. For IgG plates, dilute goat anti-mouse IgG-HRP detection antibody to a final concentration of 0.25 μg/mL in PBS with 1% milk.
  f. Seal the plates and incubate for 1 h at 25°C.
  g. After incubation, wash the plates 5 times with 200 μL of 0.05% PBST.
18. Incubate substrate and measure absorbance.
  a. Add 100 μL one-component TMB substrate to each well and incubate for 3–5 min in the dark at 25°C.
  b. Add 50 μL of stop solution to each well.
  c. Measure absorbance at 450 nm using the plate reader and Gen5 software.

***Note:*** Allow the TMB substrate solution to reach 25°C before adding it to the wells.

19. Software and datasets.
  a. Plate reader software: Gen5TM Microplate Data Collection and Analysis Software.
  b. Data analysis software: GraphPad Prism 9.0.0.

### Lenti-pseudovirus production

**Timing: 6 days**

This step involves packaging SARS-CoV-2 spike pseudoviruses based on the HIV- 1 backbone to evaluate the neutralizing antibody titers in the plasma of immunized mice.

Day 1.

20. Seed 2×10^7^ HEK 293T cells in T75 flasks to have a confluency of 90% next day at the time of transfection.

Day 2.

21. Transfection.
  a. Combine 10 μg psPAX2, 10 μg pLenti-CMV Puro-luc and 5 μg pcDNA3.1 + S.dCT13 plasmids in 1.5 mL Opti-MEM.
  b. Dilute 75 μg PEI in 1.5 mL Opti-MEM.
  c. Gently mix the diluted PEI to plasmids.
  d. Incubate the PEI-plasmids mixture for 15 min at 25°C.
  e. Carefully add the mixture to the HEK 293T cells.
  f. Replace with 15 mL of fresh complete medium 6 h post transfection.

Day 3.

22. Seed HEK 293T-ACE2 cells into 96-well plates.
  a. Aspirate the medium and wash HEK 293T-ACE2 cells twice with sterile 1 × PBS.
  b. Add 2 mL 0.05% trypsin-EDTA to the T75 flask to completely cover cells and incubated at 37°C for 2 min.
  c. Check for cell detachment under a microscope, then add 4 mL of complete medium to stop the reaction. Collect the liquid in sterile tubes and centrifuge at 500 *g* for 5 min at 25°C.
  d. Aspirate the supernatant and resuspend the cells in 2 mL of complete medium.
  e. Count cells using a cell counter.
  f. Resuspend HEK 293T-ACE2 cells in DMEM containing 5% FBS, and seed 2 × 10^4^ cells per well in 96-well plates.
  g. Place plates at the 37°C incubator with 5% CO_2_.

Day 4.

23. Collect the pseudovirus supernatant.
  a. Transfer the culture medium to a 15 mL sterile centrifuge tube 48 h post transfection.
  b. Purify pseudovirus supernatant by filtration using a 0.45 μm filter.
  c. Store the pseudovirus supernatant at 4°C for 1–2 days or at −80°C for long-term storage.

***Note:*** Avoid repeated freezing and thawing.

24. Pseudovirus titer determination.
  a. Add 50 μL pseudovirus to per well of HEK 293T-ACE2 cells, performing three replicates.
  b. Centrifuge the plates for 30 min at 1680 *g* at 25°C.
  c. Incubate at 37°C incubator with 5% CO_2_ for 36–48 h.

Day 6.

25. Luciferase luminescence measurement.
  a. Discard the supernatant after incubation.
  b. Add 100 μL of lysis buffer, and incubate for 15 min at 25°C.
  c. Add 100 μL of luciferin substrate to the lysate and allow to react for 1–2 s.
  d. Transfer 100 μL of the mixture to a white plate and measure luciferase luminescence using a luminometer.
  e. Dilute the virus based on the measured luminescence values to achieve a readout that is 100–500 times above the background.

### Lentiviral luciferase-based pseudovirus neutralization assay

**Timing: 4 days**

This step involves co-incubating the SARS-CoV-2 spike pseudovirus with plasma from immunized mice to detect the neutralizing antibody titers.^1^^,^^2^^,^^3^^,^^4^^,^^5^^,^^6^^,^^7^^,^^8^^,^^9^

**Table 3:** Plasma dilution for the neutralization assay

Sample ID	1	2	3	4	5	6	7	8	9	10	NPC	NVC
A	1:20	1:20	1:20	1:20	1:20	1:20	1:20	1:20	1:20	1:20	N/A	N/A
B	1:60	1:60	1:60	1:60	1:60	1:60	1:60	1:60	1:60	1:60	N/A	N/A
C	1:180	1:180	1:180	1:180	1:180	1:180	1:180	1:180	1:180	1:180	N/A	N/A
D	1:540	1:540	1:540	1:540	1:540	1:540	1:540	1:540	1:540	1:540	N/A	N/A
E	1:1620	1:1620	1:1620	1:1620	1:1620	1:1620	1:1620	1:1620	1:1620	1:1620	N/A	N/A
F	1:4860	1:4860	1:4860	1:4860	1:4860	1:4860	1:4860	1:4860	1:4860	1:4860	N/A	N/A
G	1:14580	1:14580	1:14580	1:14580	1:14580	1:14580	1:14580	1:14580	1:14580	1:14580	N/A	N/A
H	1:43740	1:43740	1:43740	1:43740	1:43740	1:43740	1:43740	1:43740	1:43740	1:43740	N/A	N/A

NPC, no plasma control (virus and cells only); NVC, No virus control (cells only).

Day 1.

26. Seed HEK 293T-ACE2 cells in 96-well plates.
  a. Gently aspirate the medium from the culture flask and wash HEK 293T-ACE2 cells twice with sterile 1 × PBS.
  b. Add 2 mL of 0.05% trypsin-EDTA to the T75 flask, covering the cells completely, and incubate at 37°C for 2 min.
  c. Check under a microscope for cell detachment. Add 4 mL of complete medium to stop the trypsin-EDTA reaction. Collect the liquid in sterile tubes and centrifuge at 500 *g* for 5 min at 25°C.
  d. Aspirate the supernatant and resuspend the cells in 2 mL of complete medium.
  e. Count the cells using a cell counter.
  f. Resuspend HEK 293T-ACE2 cells in DMEM containing 5% FBS, and seed 2 × 10^4^ cells per well into a 96-well tissue culture plate.
  g. Incubate plates at 37°C with 5% CO_2_.

Day 2.

27. Dilute the plasma.
  a. Add 90 μL of FBS-free DMEM to row 1 of a new 96-well round- bottom plate and 60 μL to rows 2–8. Add 60 μL of FBS-free DMEM to column 11 and 120 μL to column 12.
  b. Add 10 μL of plasma to each well in row 1 (except for columns 11 and 12), achieving a starting dilution of 1:20. Mix thoroughly.
  c. Transfer 30 μL from row 1 to row 2 and mix thoroughly. Repeat this dilution process for rows 2 through 8, discarding 30 μL from the final row (Table 3). Each 96-well plate can accommodate 10 samples.
28. Thaw the pseudovirus. Remove pseudovirus aliquots from the −80°C freezer and allow them to reach 25°C.
29. Transfer 60 μL of pseudovirus to the diluted plasma in the 96-well round- bottom microplate (except in NVC wells). Mix thoroughly.
30. Centrifuge the plates at 1680 *g* for 30 min at 20°C. Incubate the plates at 37°C with 5% CO_2_ for 36–48 h.

Day 4.

31. Luciferase luminescence measurement.
  a. After incubation, discard the supernatant.
  b. Add 100 μL of lysis buffer to each well and incubate for 15 min at 25°C.
  c. Add 100 μL of luciferin substrate to the lysate and allow to react for 1–2 s.
  d. Transfer 100 μL of the mixture to a white plate and measure luciferase luminescence using a luminometer.
32. NT50 interpolation.
  a. Use GraphPad Prism9.0.0 to interpolate the NT50 titers.
  b. Define the SARS-CoV-2 pseudovirus neutralization titers as the plasma dilution at which a 50% reduction in relative light units (RLU) is observed compared to the difference between the NVC and NPC wells.

## Expected outcomes

**Figure 2:**
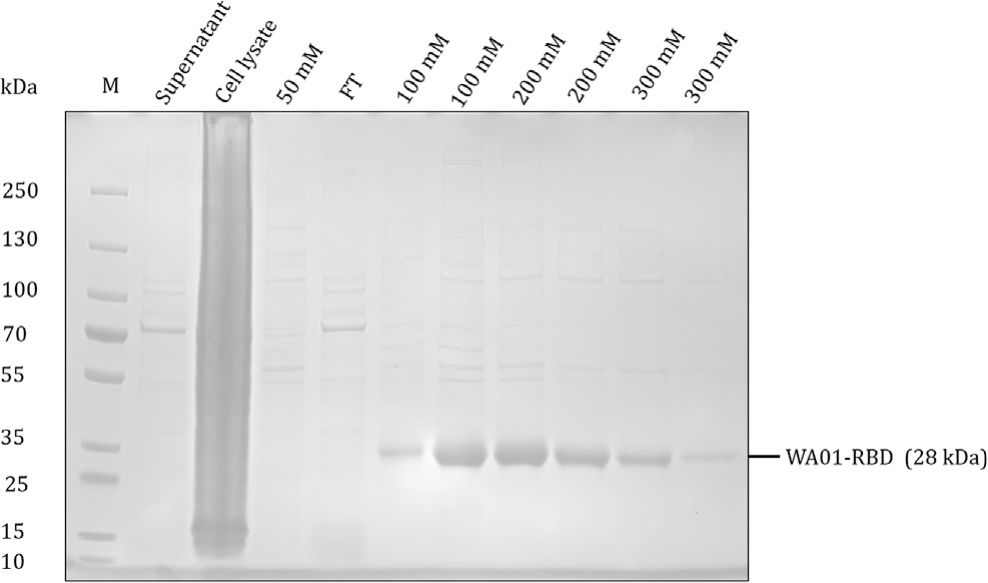
SDS-PAGE to detect WA01-RBD protein expression and purification Samples eluted with different imidazole concentrations were detected by 4%–20% SDS-PAGE.

The 239F cell cultures were centrifuged to remove cells and cellular debris, followed by incubation with Ni-NTA His-Tag Purification Agarose. A low concentration of imidazole was applied to elute non-specific proteins, while higher concentrations of imidazole (typically 100–300 mM) were used to elute the target proteins. This method was effective for the elution of WA01-RBD. The molecular weight of the primary bands was confirmed using SDS-PAGE (stained with 1% Coomassie Blue R250), with the expected size of 28 kDa (Figure 2).

**Figure 3:**
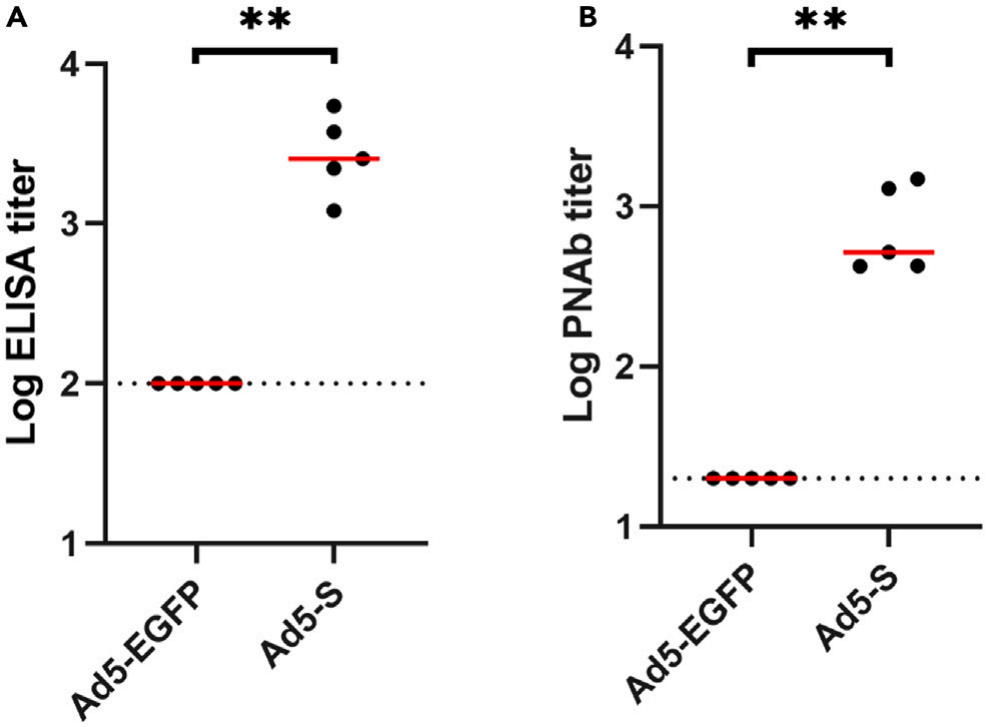
Humoral response at week 4 post-vaccination with Ad5-vectored vaccines (A) Plasma S-specific binding antibody responses were quantified by ELISA at week 4 post-vaccination. (B) Plasma neutralizing activity of elicited antibody responses were assessed at week 4 after vaccination using pseudovirus SARS- CoV-2 neutralization assays. Data were analyzed using Mann-Whitney tests: (A) *p* = 0.0079∗∗ and (B) *p* = 0.0079∗∗.

Each group of five mice was immunized with either Ad5-EGFP or Ad5-S. Four weeks post-immunization, blood was collected, and plasma was separated for the detection of S-specific binding and neutralizing antibodies (Figures 3A and 3B). Mice immunized with Ad5-S exhibited significantly higher titers of S-specific binding and neutralizing antibodies compared to the control group immunized with Ad5-EGFP.

## Limitations

Due to limited serum available from the mice, the lower limit of detection is set at 1:20. This threshold could be improved with a larger serum volume but ensures consistent and reproducible results within the constraints of this experiment.

It’s important to note that focusing exclusively on RBD-specific antibodies may not provide a complete picture of the antibody response to SARS-CoV-2. Since the RBD represents only part of the spike protein, assays utilizing the full-length spike protein might offer a more comprehensive assessment of the immune response.

Additionally, pseudovirus-based assays, while valuable, may yield results that differ from assays using live viruses or *in vivo* models. Therefore, these findings should be interpreted with caution and, if possible, compared with results from assays involving authentic viruses for greater accuracy.

## Troubleshooting

### Problem 1

High background or signal in ELISA (related to Step 18c).

### Potential solutions

If the negative control group shows a higher signal, possible causes include.

• Insufficient washing or residual solution in the wells after washing. Increase the wash solution volume to 300 μL per well. After each wash, tap the plate firmly to remove residual liquid on a clean paper towel. Repeat 3 times.
• Over-incubation. Prolonged incubation can increase background signals without improving sensitivity, as the TMB solution will react over time. Follow the recommended protocol incubation times strictly.

### Problem 2

Low or unstable signal in ELISA (related to Step 18c).

### Potential solutions

Possible causes include improper pipetting technique or the introduction of air bubbles, which affect volume accuracy. To avoid these issues, ensure proper pipetting and prevent cross-contamination between samples.

• Ensure reagents are not expired, as this may cause variable results.
• Allow the TMB color developer to reach ambient temperature before use.
• Protect plates from light during incubation steps to maintain assay accuracy.

### Problem 3

Low pseudovirus titer (related to Step 25d).

### Potential solutions

• Ensure that the plasmids used for virus production is of high quality and concentration.
• Ensure that the cells used for transfection are in good growth condition and have reached at least 90% confluency.
• Six hours after transfection, replace the medium to promote robust cell growth and higher virus yields.
• Immediately conduct the viral titer assay after harvesting the pseudovirus.
• Handle the pseudovirus carefully, avoid vigorous shaking, and minimize time at 25°C, as the pseudovirus is sensitive to physical stress and heat. Avoid repeated freeze-thaw cycles and use thawed virus immediately.

### Problem 4

Fluctuations in luminescence values during the neutralization assay (related to Step 31d).

### Potential solutions

• When performing plasma gradient dilutions, ensure precise sample additions and liquid handling.
• Avoid creating bubbles while mixing.
• When incubating the plasma-virus mixture or transduced cell plates, avoid stacking 96-well microplates to ensure even heating across all wells.
• When adding the plasma-virus mixture to the cells, do so gently to avoid detaching cells. Similarly, when discarding waste liquid during sample collection, handle with care to prevent cell detachment.

## Resource availability

### Lead contact

Further information and requests for resources and reagents should be directed to and will be fulfilled by the lead contact, Dr. Jingyou Yu (yu_jingyou@gzlab.ac.cn).

### Technical contact

Further information and requests for resources and reagents should be directed to and will be fulfilled by the technical contacts, Yao Zhang (zhang_yao2@gzlab.ac.cn) and Shixiong Li (li_shixiong@gzlab.ac.cn).

### Materials availability

This study did not generate any unique reagents.

### Data and code availability

This study did not generate/analyze any datasets or code.

## Acknowledgments

We thank the members of Yu lab for their advice, assistance, and reagents. This work was supported by the Major Project of Guangzhou National Laboratory (GZNL2023A01009 and GZNL2023A01005 to J.Y.); the National Natural Science Foundation of China (NSFC 82371831 to J.Y.); the Joint Project by Guangzhou National Laboratory and State Key Laboratory of Respiratory Disease, Guangzhou Medical University (GZNL2024B01006 to J.Y.); and the Young Talent Program of China (HJJH22-004 to J.Y.).

## Author contributions

Conceptualization, J.Y.; investigation, Y.Z., S.L., and J.Y.; writing – original draft, Y.Z. and S.L.; writing – review and editing, J.Y..; funding acquisition, J.Y.; supervision, J.Y.; Y.Z. and S.L. contributed equally to this work.

## Declaration of interests

The authors declare no competing interests.
